# *FRA2* Is a STAT5 Target Gene Regulated by IL-2 in Human CD4 T Cells

**DOI:** 10.1371/journal.pone.0090370

**Published:** 2014-02-28

**Authors:** Aradhana Rani, Roseanna Greenlaw, Manohursingh Runglall, Stipo Jurcevic, Susan John

**Affiliations:** 1 Division of Transplantation Immunology and Mucosal Biology, King's College London, London, United Kingdom; 2 Department of Immunobiology, King's College London, London, United Kingdom; University of Cologne, Germany

## Abstract

Signal transducers and activators of transcription 5(STAT5) are cytokine induced signaling proteins, which regulate key immunological processes, such as tolerance induction, maintenance of homeostasis, and CD4 T-effector cell differentiation. In this study, transcriptional targets of STAT5 in CD4 T cells were studied by Chromatin Immunoprecipitation (ChIP). Genomic mapping of the sites cloned and identified in this study revealed the striking observation that the majority of STAT5-binding sites mapped to intergenic (>50 kb upstream) or intronic, rather than promoter proximal regions. Of the 105 STAT5 responsive binding sites identified, 94% contained the canonical (IFN-γ activation site) GAS motifs.

A number of putative target genes identified here are associated with tumor biology. Here, we identified Fos-related antigen 2 (FRA2) as a transcriptional target of IL-2 regulated STAT5. FRA2 is a basic -leucine zipper (bZIP) motif ‘Fos’ family transcription factor that is part of the AP-1 transcription factor complex and is also known to play a critical role in the progression of human tumours and more recently as a determinant of T cell plasticity. The binding site mapped to an internal intron within the *FRA2* gene. The epigenetic architecture of *FRA2*, characterizes a transcriptionally active promoter as indicated by enrichment for histone methylation marks H3K4me1, H3K4me2, H3K4me3, and transcription/elongation associated marks H2BK5me1 and H4K20me1. *FRA2* is regulated by IL-2 in activated CD4 T cells. Consistently, STAT5 bound to GAS sequence in the internal intron of FRA2 and reporter gene assays confirmed IL-2 induced STAT5 binding and transcriptional activation. Furthermore, addition of JAK3 inhibitor (R333) or Daclizumab inhibited the induction in TCR stimulated cells. Taken together, our data suggest that *FRA2* is a novel STAT5 target gene, regulated by IL-2 in activated CD4 T cells.

## Introduction

Signal transducers and activators of transcription STAT5a and STAT5b (collectively called STAT5) are highly homologous proteins that are encoded by two separate genes and are activated by Janus-activated kinases (JAK) downstream of cytokine receptors. STAT proteins are activated by a wide variety of cytokines, all of which use the JAK-STAT signalling pathway as their main mode of signal transduction [1]. Upon activation by cognate JAKs, STAT proteins, dimerize and translocate into the nucleus where they bind to the promoters of genes containing the consensus recognition motif (GAS motif-TTCN_3_GAA) resulting in the transcriptional regulation of target genes.

Several studies have shown that STAT5 proteins regulate multiple genes primarily involved in T cell survival, proliferation, differentiation and homeostasis, either by transcriptional activation or repression by recruitment of negative regulatory cofactors [2]. Given its critical role in vital cellular processes, major efforts have been made to identify direct cellular targets of STAT5 using techniques such as ChIP-chip and ChIP-seq techniques [3], [4], [5]. However, the target genes identified by STAT5-ChIP differ between cell types and are further influenced by cell treatments and time points studied [6], [7]. Thus, the range of target genes that STAT5 regulates may differ from one cell to another, from one cell treatment to another as well as being dependant on the time point studied. Nevertheless, these studies have begun to provide important mechanistic insights into the regulation of various biological and cellular processes by STAT5.

In this study, we aimed to identify genes regulated by IL-2/STAT5 in preactivated CD4 T cells by ChIP, with a view to understanding the range of STAT5 target genes and the molecular actions regulated by IL-2 in this cell type. Analysis of the target sites provided an insight at various levels, such as relative positioning with respect to the transcription start site (TSS) of genes, with only a small percentage (11%) within 10 kb of the TSS of gene/s; presence/absence of GAS sequences, which revealed that 94% contained the consensus/non-consensus recognition motif; epigenetic changes associated with mapped sites; identification of putative downstream-target genes and hence the potential cellular processes and biological pathways that may be regulated by STAT5.

Previously, it was shown that IL-2 and STAT5 plays a prominent role in human and murine TH2 cell differentiation and we recently showed that *c-maf* is an IL-2 induced STAT5 target gene that is involved in this process [4], [8], [9], [10]. In keeping with the Th2 theme for IL-2/STAT5, here we present the characterization of the FRA2 as a STAT5 target gene, identified from the ChIP cloning studies. In the immune system, FRA2 is involved in IL-4 gene regulation and is involved in CD4-Th2 cell differentiation [11]. More recently, FRA2 has been documented as a key determinant of cellular plasticity during CD4 T cell differentiation [12]. FRA2 is a member of the FOS/JUN subgroup of bZIP transcription factors (TFs) and the AP1 transcription factor complex, which consists of heterodimers formed by the Fos family (c-FOS, FOSB, FRA1, and FRA2) with Jun family (c-JUN, JUNB and JUND) of transcription factors [13], [14]. AP1 complexes are able to activate or suppress the expression of many genes involved with proliferation, differentiation, and survival: functions in common with STAT5. Consistent with its role in vital cellular functions, dysregulated expression of FRA2 has also been associated with lymphomas [15].

In this study, we identify one of the ChIP-cloned sequences to map to an internal intronic region of the FRA2 gene, and show that *FRA2* is induced by T –cell receptor activation in human CD4 T cells in an IL-2/JAK3/STAT5 dependent manner.

## Materials and Methods

### Human blood CD4 T cell isolation and culture conditions

PBMCs were isolated by density gradient centrifugation from heparinized buffy coat obtained from the National Blood Service (St. George's Hospital, London). CD4 T cells were isolated by positive selection from the PBMCs using CD4 microbeads and (Miltenyi Biotec, Auburn, CA) an LS column as described in the manufacturer's instructions. Cells were maintained in RPMI 1640 medium (PAA Laboratories, Yeovil, UK) with 10% fetal bovine serum (FBS, PAA laboratories), 2 mM L-glutamine and penicillin-streptomycin (100 IU/ml and 100 µg/ml, Sigma-Aldrich, UK). The freshly isolated CD4 T cells were either used immediately or activated with PHA 2 µg/ml (EY Laboratories, San Mateo,CA) for 72 hours to generate PHA blasts. The PHA blasts were rested for 16 hours in complete RPMI 1640 medium before restimulation. Where appropriate, cells were stimulated with 100 U/ml IL-2 (TECIN, [Teceleukin]; Roche) for 20 minutes or as stated for activation kinetics experiments. In other experiments, CD4 T cells were activated with plate bound anti-CD3 (UCHT1; 10 µg/ml) and soluble anti-CD28 Ab (2 µg/ml; Ancell, Bayport, MN) with or without the JAK3 inhibitor R333 (5 µM; a gift from Rigel Pharmaceuticals, South San Francisco) or humanized anti-Tac (HAT, or Daclizumab (Zenapax), 10 µg/ml, Roche, Nutley, NJ).

### Chromatin Immunoprecipitation, Cloning and Sequencing

Chromatin immunoprecipitation (ChIP) cloning experiments were performed using 1.5×10^7^ CD4 T cells stimulated with IL-2 for 20 min followed by a 10-min crosslink with formaldehyde (0.37%) at 37°C and immediate termination of the reaction by the addition of 1.25 M glycine, as previously described [6]. The cells were harvested and shearing of the chromatin was done on a Misonix 3000 sonicator. The sonicated chromatin was immunoprecipitated for an hour using µMACS protein A microbeads (Miltenyi Biotec, UK) and anti-STAT5a and -STAT5b monoclonal antibodies or control mouse IgG Abs (all from Zymed Laboratories, UK). Purified chromatin was blunt-end repaired using T4 DNA polymerase (New England Biolabs, UK), ligated to linkers, PCR amplified, and cloned into TOPO vector (Invitrogen, UK) and sequenced (Cogenics, UK). Validation and confirmation of the GAS sequence within the *FRA2* gene, as a STAT5 target was done by quantitative PCR reaction, performed as described in the LowCell# ChIP Kit TM (Diagenode, UK) and data interpreted as a percentage of the starting material (Input).

### *In silico* mapping and screening of cloned sequences

Individual clones were blasted against the UCSC Genome Browser (http://genome.ucsc.edu) and the public databases at NCBI using the BLAST tool (http://www.ncbi.nlm.nih.gov/) and filtered for 100% identity.

The sequences were screened for closest genes and conservation tracks using the March 2006 (hg 18) human reference sequence (NCBI Build 36.1). Additionally, the sequences were mapped to the whole genome analysis of DNase-I Hypersensitivity sites in CD4 T-cells using the May 2004 (hg17) human reference sequence (NCBI Build 35) [16]. Gene ontology analyses of all the cloned sequences were performed using the PANTHER database [17].

### RNA isolation, cDNA synthesis and RT-PCR

Total RNA was extracted using TRIzol reagent (Invitrogen Life Technologies, CA, USA). 500 ng of total RNA was reverse-transcribed to cDNA using the TaqMan Reverse Transcription kit (Life Technologies) following manufacturer's instructions. The first-strand cDNA obtained using the above protocol was amplified using different primer/probe mix in 384-well plates using the ABI Prism 7900 HT Sequence Detection System (Applied Biosystems, Foster City, CA). The sequence detector SDS 2.3 software (Applied Biosystems) was used to export the Ct values. 18S gene expression was used as the endogenous reference gene. Quantification was done by applying the following equation: RQ = 2^−ΔCt^ where RQ represents the estimated amplification assuming a doubling of material during each PCR cycle and ΔCt is the Ct(gene)-Ct(18 s) and Ct is the cycle at which the threshold is crossed.

### Cell transfection and luciferase reporter assays

HEK293T cells were transfected to reconstitute IL-2R signalling using the calcium phosphate method, as previously described [18]. Trimerized GAS1 sequence was synthesized (Sigma, UK), cloned into pGL4.23 (luc2/minP) vector (Promega, UK), and verified by sequencing (Eurofins MWG-Operon, Germany). 2 µg of IL-2 receptor β chain, 0.5 µg of common cytokine receptor γ chain, 0.25 µg of JAK3, and 75 ng of each of the Stat5 plasmids, 0.5 ng of the control pTK-Renilla Lucifearse plasmid and 1 µg of the reporter plasmids were cotransfected into 293 T cells. After 24 hours, cells were treated with 100 IU of IL-2 overnight and assayed for luciferase activity. Luciferase assays were performed by using the Dual-Glo luciferase kit (Promega, UK) according to the manufacturer's instructions. To normalize for transfection efficiency, luciferase activity was divided by the Renilla luciferase activity, which gave a relative luciferase activity for each sample. Further, the IL-2 induced value is divided by the uninduced value to give a fold induction.

## Results and Discussion

### Identification of STAT5 regulated genes

In vivo STAT5 targets in human activated CD4 T cells were identified by ChIP, followed by cloning and sequencing. A total of 105 unique binding sites were mapped and assigned to the genome (Table S1). The genomic distribution of STAT5 binding sites were analysed with respect to an annotated gene as being present within introns, or, <10 kb upstream of the gene and in intergenic regions (here defined as regions >10 kb from the 5′ end of the two closest annotated genes on a chromosome). Thus, for STAT5 target sites located <10 kb upstream of a gene, the closest gene was considered as a potential regulatory target. For fragments mapping to an intron, the corresponding gene was designated the potential STAT5 target gene and for intergenic binding sites, both the genes involved were considered as potential targets.

**Figure 1:**
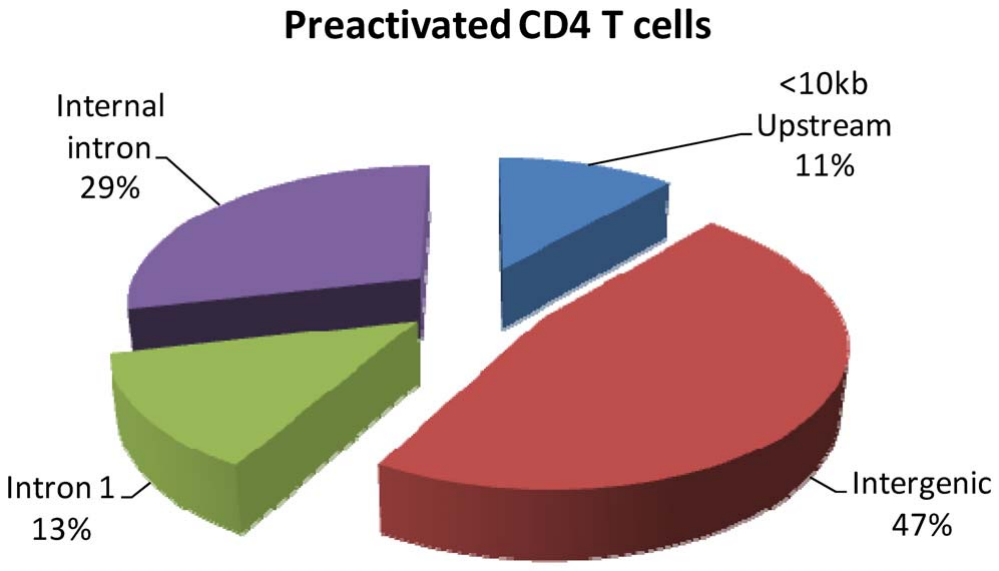
The locations of putative STAT5 binding sites in activated CD4+ T cells. Mapping of STAT5 binding sites relative to annotated genes were categorized into four groups. Intergenic region denotes binding sites present greater than 10<10 kb upsteam denotes the region less than 10 kb upstream of the 5′ region of the nearest gene. Internal intron denotes introns other than intron 1 of the gene and intron 1 depicts the presence of a binding site within the first intron of the gene.

Of the 105 unique cloned inserts isolated from activated CD4 T cells, greater than one third (42%) of the fragments lie within introns of annotated genes, with a larger percentage within intron 1 (13%). A significant number of binding sites (49, 47%) correspond to intergenic regions, while 12 of the fragments (11%) were within 10 kb upstream of a gene (Figure 1). These findings predict that generally STAT5 binds within enhancer rather than promoter regions and facilitates transcription through long distance interactions by DNA looping [19]. ChIP-seq studies on STAT1 and NF-kB are consistent with these findings and demonstrate that the percentage of binding sites in intergenic or intronic regions far exceeds those in the promoters of genes [20], [21]. Studies of other transcription factors have shown that binding sites can be as far as 168 kb and 206 kb upstream of a transcription start site [22].

**Figure 2:**
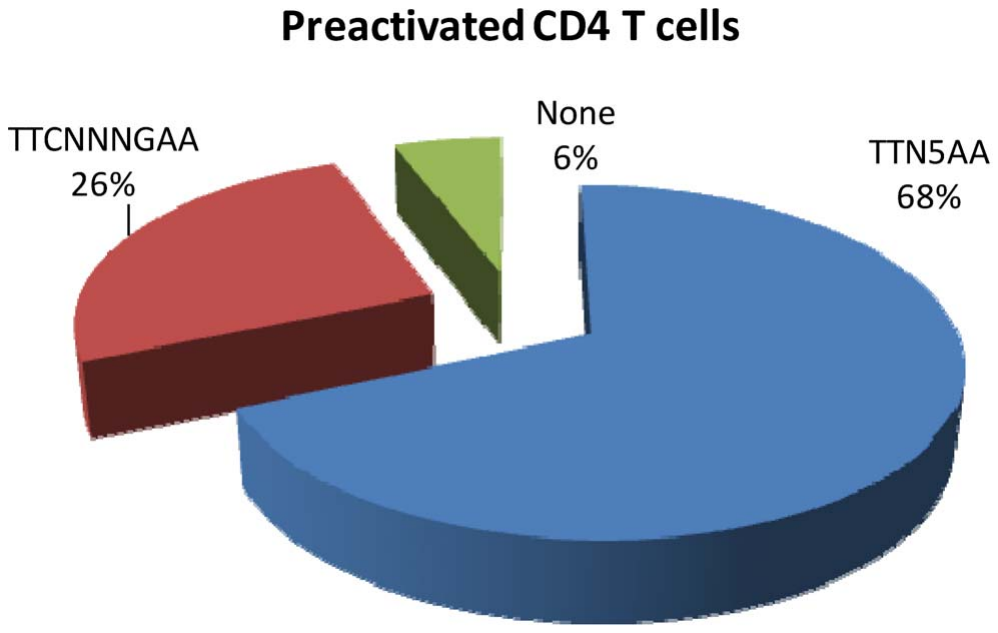
STAT5 motif analysis of the mapped ChIP clones in activated CD4+ T cells. Out of the 105 binding sites for activated CD4+ T cells immunoprecipitated with anti-STAT5 Ab, 68% had TTN_5_AA motifs and TTCN_3_GAA sites were present in 26% of the target sites. The labels denote the category name and percentage.

Further, an *in silico* analysis of the clones was performed to investigate the presence of STAT5 recognition motifs TTCN_3_GAA and TTN_5_AA. The consensus GAS recognition motif for dimeric STAT5 binding is TTCN_3_GAA [23]. However, STAT5 can also potentially recognize the more general consensus TTN_5_AA, when it binds DNA as a tetrameric complex of N-domain linked dimers [23]. The motifs were subdivided into three groups: clones containing TTCN_3_GAA sites, clones containing TTN_5_AA sites and those containing no GAS motifs. The results are summarized as a pie-chart in Figure 2. The striking observation for this analysis was that the majority of the sequences contained the more general TTN_5_AA sequence, rather than the consensus GAS motif, TTCN_3_GAA, perhaps indicating a less stringent requirement for DNA recognition, reminiscent of DNA binding by tetrameric STAT5 complexes. Several clones had multiple GAS motifs of either type. In view of this observation, it is interesting to note previous studies that have shown strengthening by virtue of the protein-protein interactions mediated via the N-domains, whereby tetrameric STAT5 complexes can specifically bind to tandem-linked non-consensus or non-GAS motifs in addition to consensus GAS motifs, thus increasing the repertoire of STAT5-regulated genes [18]. Two other STAT5-ChIP-cloning studies have also observed use of only the TTN_5_AA sequences [7], while studies of other transcription factors have noted the use of similar non-consensus recognition motifs [24]. These findings are consistent with the recent demonstration that the STAT5 tetramer plays a critical role in the cytokine responses of normal immune function and in the resistance to malignant transformation of cells [25], [26]. Consistent with previous observations from in-vitro STAT5 binding studies, a small number of the putative STAT5-binding sites did not contain a recognizable GAS motif (23). Similar results have been obtained for other transcription factors and other STAT5-ChIP studies where it was observed that the frequency of factors not binding to consensus sequences was very high [24]. Taken together, STAT5 appears to favor the less stringent general consensus GAS motif, for *in-vivo* DNA binding, which would potentially be bound by tetrameric STAT5.

Although an in-silico analysis of the target binding sites enhanced our knowledge of potential STAT5-regulated genes, they are in need of experimental validations and functional studies. Verification of the robustness of the ChIP assay was confirmed by independent ChIP-PCR analysis on the target sites which demonstrated that they were bonafide STAT5 binding sites.

**Figure 3:**
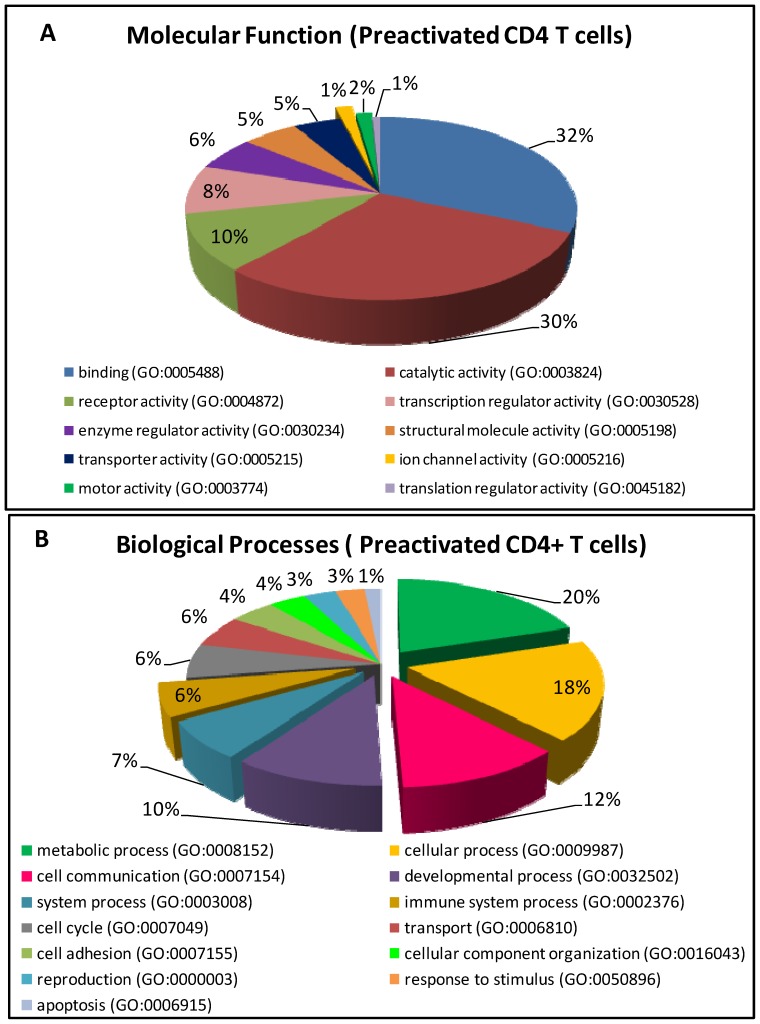
Gene ontologies of putative STAT5 targets from activated CD4+T cells. Gene function of putative binding sites is based on the summary information provided in the PANTHER database. Out of the 105 binding sites identified, functional categorisation was done for 104 genes, out of which 99 could be classified and the remaining 5 genes were of unknown/uncategorized function. Those 99 genes were classified based on their molecular function (A) and biological processes (B). In some instances, a given gene was represented in more than one category (for example, FRA2 was incorporated under the binding (GO:0005488) as well as transcriptional regulator activity (GO: 0030528) molecular functions).

Although the genes associated with the putative STAT5 binding sites have not all been confirmed as being bonafide STAT5 regulated genes, they were classified into functional categories to gain insight into the cellular functions that could potentially be regulated by STAT5 in activated CD4+ T cells. One important caveat to this analysis is that, where a particular binding site could not be annotated to a single gene, as in the case of intergenic sites, both flanking genes were considered in the functional classification. They were categorized using the PANTHER database (Figure 3A and 3B). The functional categories predicted from this analysis included metabolic, cellular, cell communication, developmental, immune system, cell cycle, transport and apoptosis. While many of the predicted functions from this analysis are consistent with known functions of STAT5, for instance cell cycle, differentiation, survival, signal transduction, others such as cell adhesion, transport and metabolism are novel functions predicted from this study.

The molecular functional categories of target sites that were most highly represented in activated CD4+ T cells were genes encoding binding, catalytic activity, receptors, transcription, enzymes and proteins involved in cell differentiation, proliferation and survival. Consistent with the known association of aberrant constitutive activation of STAT5 with human cancers/tumours of haematological and non-haematological origins, several potential oncogenes were identified as putative downstream target genes. These include, *TLE4* [27], *ACSL6* [28], *TOP1* [28], *TCEA1*, *TSC22D1* [29], *KIF1A* [30], *HERC2* [30], *LRP1B* [31], *CDH10* [32], [33], *ST6GAL2* [32], *FAT1* [33], *CHD1* [34], *GRID1* [35], [36], *IGFBP3* [35], *ADAM22* [37], *CHD6* [38] and and *FRA2* [15]. Thus, the functional classification of potential STAT5-regulated genes confirmed known functions of STAT5 and furthermore provided novel insight into potential unknown cellular functions and oncogenes regulated by these proteins. Of these, we chose to further characterize FRA2 in this study, as this gene seemed particularly interesting in light of the fact that FRA2 is not only associated with malignant transformation, but is also a key regulator of CD4 T cell differentiation, both of which are known activities of STAT5 [11], [15].

Interestingly, *FRA2* was also identified in a STAT5-ChIP study from IL-3 stimulated BAF3 cells, suggesting that it is an important downstream mediator of STAT5 function, irrespective of the activating cytokine [39].

### *In Silico* analysis of *FRA2* locus in CD4+ T cells

**Figure 4:**
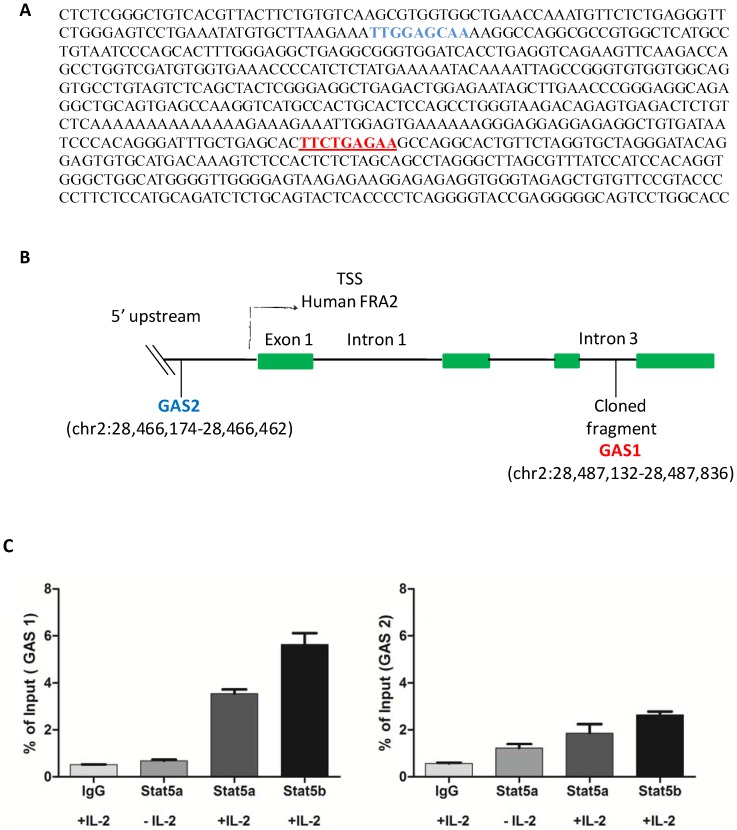
Study of the binding site, location and validation of the cloned fragment obtained by ChIP. A. Sequence of the cloned fragment and the corresponding typical and atypical GAS sequences present. There is a TTN_5_AA (blue font) sequence and one typical GAS TTCN_3_GAA (red font) sequence present within the cloned fragment. B. The relative location of the cloned fragment, GAS1 and GAS2 with respect to the FRA2 transcription start site (TSS) and intron/exon is shown schematically and its coordinates on the UCSC genome browser. Figure 4C: IL-2 induces in-vivo binding of STAT5 to GAS1 within the third intron of the FRA2 gene in preactivated CD4 T cells. STAT5-ChIP samples from ±IL-2-treated preactivated CD4 T cells were analysed by real-time PCR for specific binding to the two GAS motifs identified above. ChIP assay was performed using the LowCell# ChIP kit and ±IL-2-treated preactivated CD4 T cells. Enrichment of GAS 1 was higher when compared to GAS 2 and more so with the STAT5b antibody.

The cloned sequence that mapped to an internal intron within the *FRA2* gene contained a consensus GAS sequence, TTCTGAGAA and one other TTN_5_AA sequence: TTGGAGCAA (GAS1, Figure 4A).

To further explore the mechanism of *FRA2* regulation by STAT5, the 5′ upstream regulatory region was scanned for GAS sequences located either within or near to hypersensitive sites, identified by the CD4 DHS study [40]. One additional DHS site was identified, which contained a GAS motif (GAS2). A schematic figure representing the GAS sequences studied is shown (Figure 4B). While GAS1 represents the sequence present within the identified clone in intron 3 of the FRA2 gene, GAS2 represents the scanned GAS sequence present within the 5′ TSS. GAS 1 mapped to chromosome 2 at +17850 to +18554 bp (Intron 3) and GAS2 mapped to chromosome 2 at −3108 to −2820 bp upstream (TSS) of the FRA2 gene, both of which contained a consensus GAS motif.

To determine whether the *FRA2* GAS (GAS1 and GAS2) sequences within the cloned site and promoter region are direct targets of STAT5, quantitative real-time ChIP-PCR was performed on chromatin obtained by ChIP of IL-2-stimulated (or untreated control) PHA-activated human CD4 T cells. Specific primers encompassing the GAS fragments were used and the ChIP was performed using an anti-STAT5 antibody or IgG control. Enrichment of both GAS1 and GAS2 was observed, although the binding specificity was noticeably higher for the cloned GAS1 site, which also shows a greater enrichment for STAT5b as compared to STAT5a (Figure 4C). Enrichment of the STAT5 binding site was observed for chromatin that was immunoprecipitated with STAT5a and STAT5b antibody in the presence of IL-2 when compared to unstimulated and IgG control. Thus, IL-2 induces specific binding enrichment of STAT5b primarily to a region in the intron of the *FRA2* locus.

**Figure 5:**
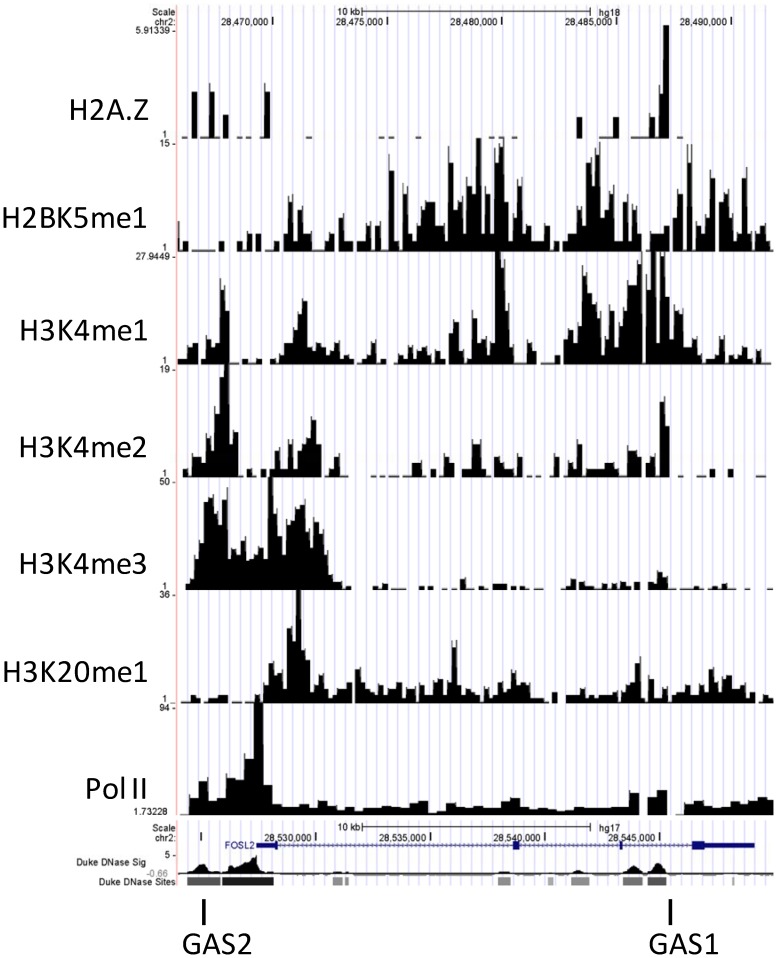
Custom Track View of DNase I hypersensitive sites (DHS) sites. DHS sites, Pol II, H3K20me1, H3K4me3, H3K4me2, H3K4me1, H2BK5me1 and H2A.Z along the FRA2 gene on Chr 2 were obtained from the UCSC Genome Browser.

The FRA2 cloned fragment, when queried for its location with respect to the genome-wide DNase HS (DHS) sites in CD4+ T cells (UCSC Genome Browser on Human May 2004 Assembly, hg17) [40], demonstrated that it is located within a DNase hypersensitive site. Genome wide studies into histone modifications by acetylation and methylation on CD4 T cells has provided valuable insights into the activation, repression or silencing of genes in these cells [41], [42]. Acetylation of histones, which is invariably associated with gene activation, is regulated by various methylations (position and state) of the lysine and arginine residues on histone tails to dictate either an activation or repression of genes [43], [44], [45]. Some of the methylation marks associated with activation are H2BK5me1, H3K4me1, H3K4me2, H3K4me3, and H4K20me1. A study of the methylation pattern available on the UCSC genome browser also suggests *FRA2* is a transcriptionally active gene in CD4+ T cells (Figure 5). The *FRA2* gene also contains regions of enrichment of the H2 variant, H2A.Z, which is often associated with nucleosome instability (Figure 5)(43). This would indicate that the chromatin in the *FRA2* locus is transcriptionally active, because the GAS motifs are located within DHSs [40]. Thus, this locus appears to be “poised” for transcriptional activation in unactivated CD4 T cells.

Mining for STAT5 target sites on the FRA2 gene was done using massive parallel sequencing (ChIP-Seq) data already available, which defines the genome-wide targets of STAT5A and STAT5B in CD4+ T cells. The data is available via the Gene Expression Omnibus (GEO), accession number GSM671400 and GSM671402 at www.ncbi.nlm.nih.gov/geo. Peaks for STAT5b binding to a region within intron 3 of the FRA2 gene was observed at chr2:28,485,399-28,485,970 on the UCSC genome browser (Human Mar. 2006 (NCBI36/hg18) Assembly). This region is located close to GAS1 identified in this study which maps to the genome base position of Chr2:28,487,132-28,487,836. The site identified by the ChIP-seq study and the present study are almost 1200 bp away from each other and it is possible that DNA binding sites were juxtaposed by DNA looping to functionally interact and orchestrate transcriptional activation.

### *FRA2* is expressed in activated CD4+ T cells and is regulated by IL-2

**Figure 6:**
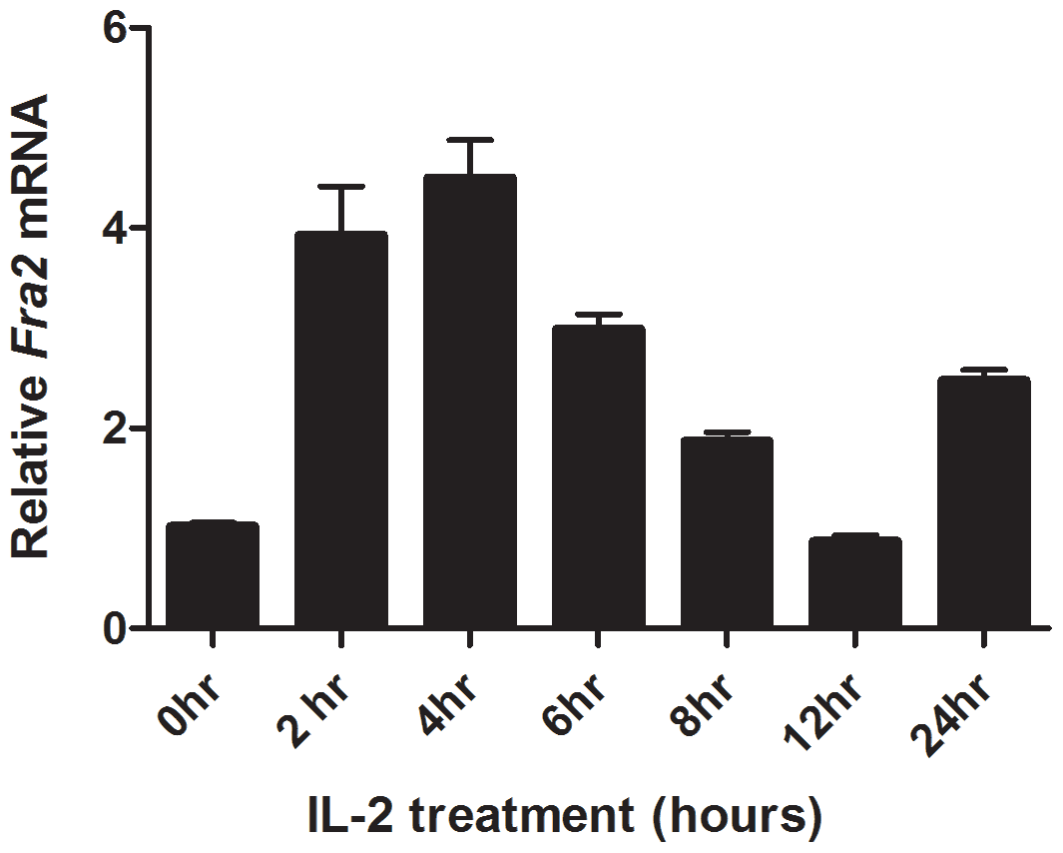
IL-2 regulates expression of FRA2. Quantitative RT-PCR was performed on PHA activated CD4+ T cells stimulated with or without IL-2 for different times to evaluate the expression of FRA2. Expression levels are presented as fold increase (logarithmic scale) and compared to the baseline levels (cells not treated with IL-2). 18 s was used as the housekeeping gene and served as the endogenous control. IL-2 stimulation strongly induced *FRA2* expression with two peaks observed at 4–6 hours and 24 hours post stimulation. This is representative of at least three independent experiments performed in triplicate.

To investigate whether IL-2 can induce *FRA2* gene expression, CD4 T cells were preactivated with PHA to achieve maximal IL-2 responsiveness by expressing the high affinity IL-2 receptor, followed by treatment with IL-2 for various times (2–24 hours) or left untreated before RNA was extracted and *FRA2* expression was analysed by quantitative real-time PCR (qRT-PCR). These studies revealed that *FRA2* is induced in a biphasic manner by IL-2 in activated CD4 T cells: with peak expression at approximately 4 hours, decreasing to basal levels within 12 hours and with a second peak at 24 hours (Figure 6). Thus, exogenous IL-2 alone efficiently stimulates *FRA2* expression in the presence of TCR stimulation.

### TCR plus CD28-mediated induction of *FRA2* is dependent on IL-2R signaling

**Figure 7:**
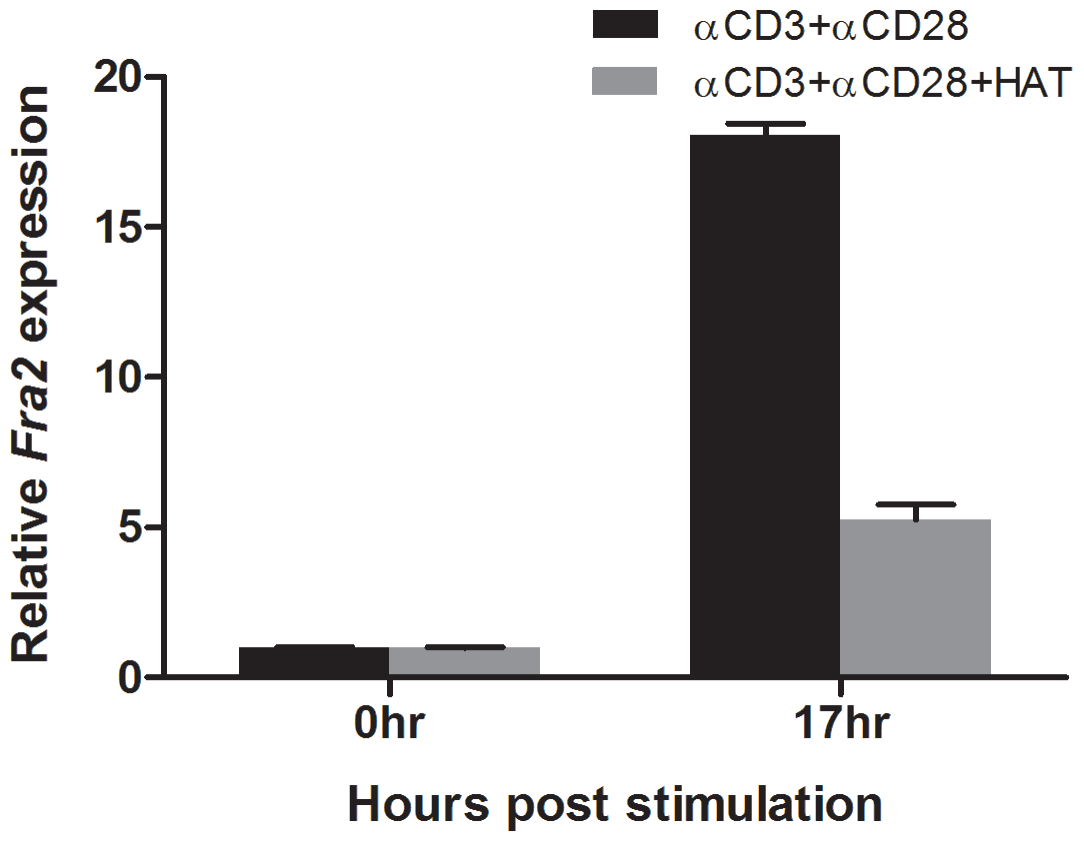
TCR induced FRA2 expression is dependent on IL-2 signaling. CD4+ T cells were TCR activated ±HAT (humanized anti-Tac antibody) treatment and mRNA was prepared pre- and 17 hours post-stimulation. qRT-PCR analysis for FRA2 expression was carried out. TCR activation induces FRA2 expression, which is abrogated by the addition of HAT, indicating that IL-2R function is essential for induction of FRA2. This is representative of at least three independent experiments performed in triplicate.

To dissect the contribution of IL-2 receptor signaling to the TCR-induced expression of *FRA2*, the effect of clinically-important inhibitors of CD25 and JAK3 on this process were examined. Cells were stimulated with anti-CD3 and anti-CD28 antibody, for specific T cell receptor activation which leads to IL-2 production. The human interleukin 2 receptor alpha (IL2Rα) is also known as CD25 and HAT blocks IL-2 signalling. Human CD4+ T cells were isolated from buffy coats and stimulated with anti-CD3 and anti-CD28 antibodies for 17 hours in the presence or absence of humanized anti-Tac (HAT) antibody (Daclizumab, anti-CD25). It has previously been shown that HAT treatment efficiently blocks JAK3/STAT5 activation and consequent production of Th1 and Th2 cytokines following anti-CD3 and anti-CD28 antibody stimulation of PBMC [46]. Here TCR activation induced *FRA2* expression 17 hours after stimulation, which was abolished in the presence of HAT. Thus, the TCR induced activation of *FRA2* is dependent on IL-2/IL-2R signaling (Figure 7).

**Figure 8:**
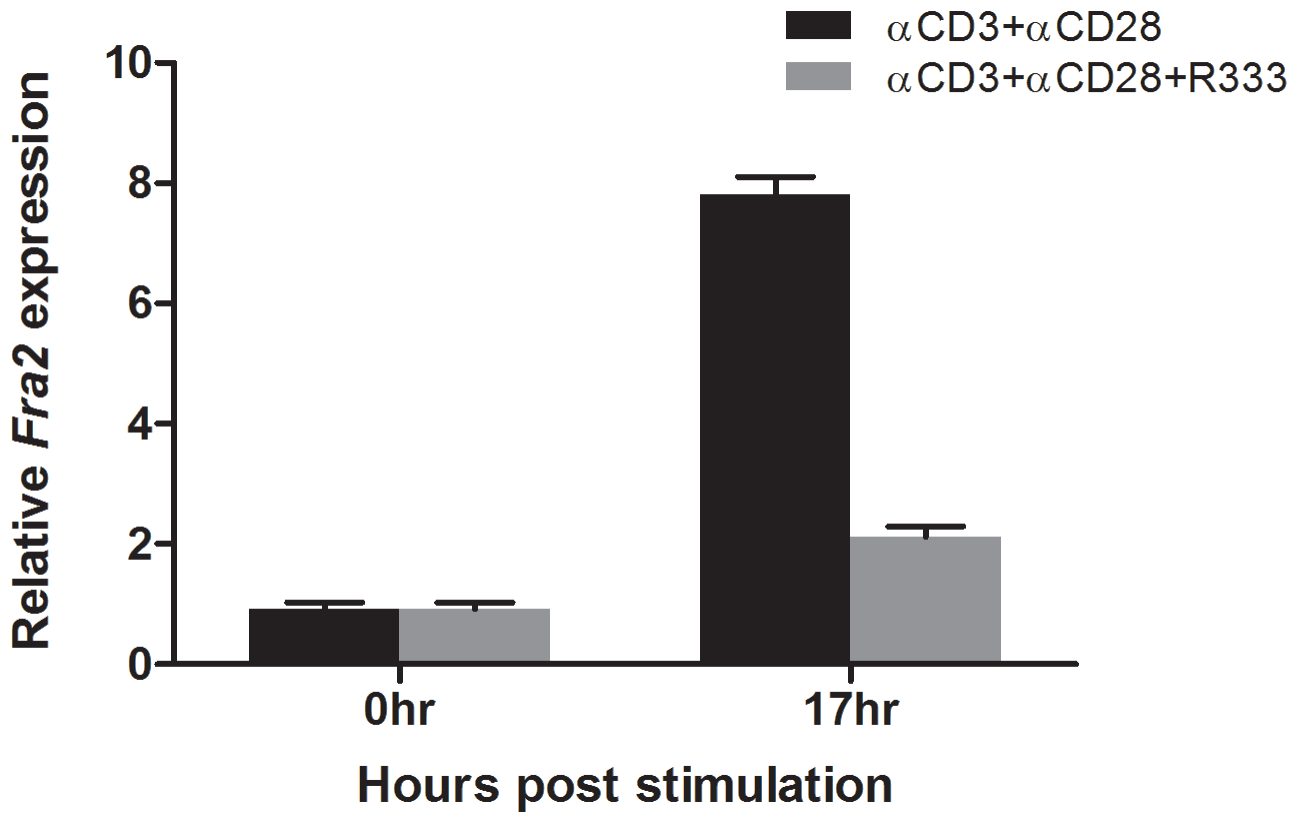
Activation of the JAK3-STAT5 pathway is essential for the induction of FRA2 gene expression by TCR activation. CD4+ T cells were stimulated via the TCR ± the JAK3 inhibitor R333, RNA was prepared four hours post treatment. qRT-PCR analysis was performed to detect the relative expression of FRA2. FRA2 gene expression was induced by TCR activation and abrogated by R333 treatment. Shown is a representative experiment performed in triplicate and repeated three times.

To confirm that induction of *FRA2* by T cell activation was specifically due to the activation of the JAK3/STAT5 pathway, a JAK3 inhibitor R333 [47], was employed during TCR activation. CD4 T cells were purified and stimulated with anti-CD3 and anti-CD28 antibodies in the presence or absence of R333. RNA was prepared from the samples and analysed by real-time PCR for *FRA2* expression. The data showed that TCR-mediated activation of CD4 T cells induced *FRA2*, 17 hours post stimulation, which was abrogated in the presence of the JAK3 inhibitor (Figure 8). Taken together, the data indicate that TCR-induced upregulation of *FRA2* gene expression is dependent on IL-2-signaling in CD4 T cells.

### STAT5 binding site, GAS1 exerts regulatory activities

**Figure 9:**
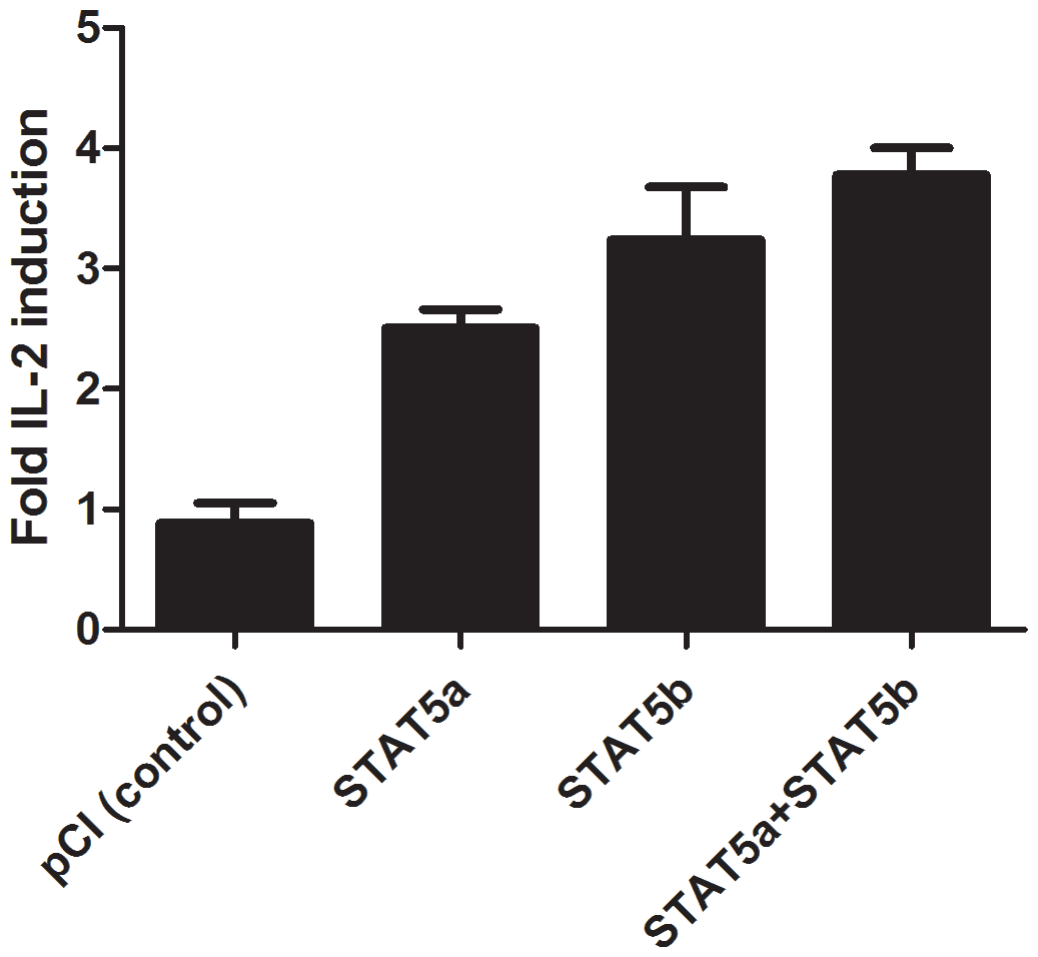
Transcriptional activities of the GAS1 sequence. Trimerised GAS1–luciferase reporter was cotransfected with IL-2R components in 293T cells, as described in materials and methods. The results are from three independent experiments performed in triplicate. This shows that the GAS1 sequence identified by the ChIP studies can be specifically activated by STAT5 in an IL-2-receptor reconstitution assay in 293T cells.

To confirm that the GAS1 sequence can induce STAT5-dependent transcriptional activity, a luciferase reporter assay was performed using an IL-2R reconstitution system, as previously described [18]. These studies revealed that IL-2-induced luciferase expression from a GAS1 driven reporter construct could be activated by transfection of STAT5a or STAT5b alone when compared to the empty vector transfected negative control, which shows no IL-2 inducible transcriptional activation of luciferase activity (Figure 9). Interestingly, consistent with the ChIP-PCR results, STAT5b was able to induce higher levels of luciferase activity as compared to STAT5a. Co-expression of STAT5a and STAT5b did not significantly differ from the results obtained with either STAT5a and STAT5b alone. These data confirm those from previous studies on optimal GAS binding sites for STAT5, which reveal a favoured consensus sequence of TTC(C/T)N(G/A)GAA [23], [48], [49], as in GAS1 and are also consistent with the relative STAT5 binding enrichment for the two GAS motifs by ChIP (Figure 4C). Taken together the findings show that IL-2 induces specific STAT5 binding and transcriptional activation from an intronic GAS motif of the *FRA2* gene.

## Conclusion

In this study, ChIP was used to generate a library of STAT5 regulated genes from activated human CD4 T cells stimulated with IL-2. Analysis of 105 putative STAT5 binding sites isolated, for their location with respect to TSS of genes, showed that the majority of the sites mapped to introns and intergenic regions, which is consistent with the findings of whole genome analysis of DNAse-1 hypersensitive sites [50]. Although the consensus GAS recognition motif for dimeric STAT5 binding is TTCN_3_GAA [23], a large proportion of the sites contained the more general consensus TTN_5_AA. Gene annotation studies predicted a number of cellular functions regulated by STAT5, many of which were previously known but also exposed novel functions such as those related to metabolism and transport. The main disease prediction from the analysis of these STAT5-target genes is cancer, which correlates with previous studies of malignancies [51], [52], [53], [54].

FRA2 is an example of one such tumor associated gene, whose regulation by STAT5 was studied here. The intronic STAT5 binding site in *FRA2* contained a consensus motif, TTCN3GAA and was located in an architectural region of chromatin that is poised for transcriptional activation. Previous studies have shown that FRA2 is expressed at the protein level during early Th2 cell polarization [11], and that FRA2 is a putative STAT5 target gene [39]. Our studies extend these observations to reveal that the FRA2 locus is in a poised chromatin state, enabling expression early during T cell activation in an IL-2 dependent manner. Interestingly, IL-2/STAT5, like FRA2 is known to inhibit Th17 differentiation, and therefore the characterization of FRA2 as a STAT5 target gene in this study may be one mechanism by which STAT5 can negatively regulate TH17 differentiation. Its role in T cell differentiation needs to be studied further.

## Supporting Information

Table S1 **Mapping of STAT5 ChIP clones.** Clones obtained from the libraries were mapped using to their Chromosomal location using the UCSC genome browser.
(XLSX)
